# Cervical Ectopic Pregnancies—Imaging and Endovascular Treatment

**DOI:** 10.3390/diagnostics15151956

**Published:** 2025-08-04

**Authors:** Maciej Szmygin, Bartosz Kłobuszewski, Karolina Nieoczym, Weronika Dymara-Konopka, Sławomir Woźniak, Hanna Szmygin, Łukasz Światłowski, Krzysztof Pyra

**Affiliations:** 1 Department of Interventional Radiology and Neuroradiology, Medical University of Lublin, Jaczewskiego 8, 20-954 Lublin, Poland; klobuszewskib@gmail.com (B.K.); lukasz.swiatlowski@umlub.pl (Ł.Ś.); krzysztof.pyra@umlub.pl (K.P.); 2 Department of Oncology, Medical University of Lublin, Jaczewskiego 8, 20-954 Lublin, Poland; karolinanieoczym1@gmail.com; 3 Department of Obstetrics and Perinatology, Medical University of Lublin, Jaczewskiego 8, 20-954 Lublin, Poland; weronika.dymara@gmail.com; 4 Department of Gynecology, Medical University of Lublin, Jaczewskiego 8, 20-954 Lublin, Poland; slavwo7572@gmail.com; 5 Department of Endocrinology, Center of Oncology of the Lublin Region St. Jana z Dukli, 20-090 Lublin, Poland; koszelh@gmail.com

**Keywords:** ectopic pregnancy, cervical, embolization, endovascular

## Abstract

**Objective**: Cervical pregnancy (CP) accounts for less than 1% of all ectopic pregnancies. The standard of management for CP is still under detailed investigation; however, among the known treatment methods, super-selective uterine artery embolization (UAE) and the use of methotrexate (MTX) have emerged as effective and minimally invasive options in recent years. Our aim is to present our center’s experience and provide available evidence evaluating the efficacy of UAE in the treatment of CP. **Materials and Methods:** This single-center and retrospective study evaluated the procedural and clinical outcomes of patients with CP who underwent endovascular uterine embolization with MTX between 2017 and 2024. Both procedural and clinical efficacy and safety, as well as the rate of complications and long-term outcomes, were noted. **Results:** A total of nine patients were diagnosed with CP (imaging examination included transvaginal ultrasound and/or magnetic resonance imaging) and referred for endovascular treatment. The mean age of the patients was 36.7 years, and the mean gestational age on admission was 9 weeks. In all cases, selective catheterization of supplying vessels and subsequent embolization with a mixture of methotrexate and gel sponge was carried out. The technical success rate was 100% with no complications. Follow-up ultrasound confirmed the disappearance of the flow signal around the intracervical gestational sac in all cases. **Conclusions:** In conclusion, this retrospective study demonstrated the procedural and clinical safety and efficacy of uterine artery embolization in patients with cervical pregnancy. This is why endovascular therapy should be proposed to these individuals and be included in treatment options discussed during multidisciplinary boards.

## 1. Introduction

Cervical pregnancy [CP] accounts for less than 1% of all ectopic pregnancies and is therefore one of the rarest types of abnormal implantation—its estimated prevalence is approximately 1 in 9000 pregnancies [1]. The etiology of this condition is the implantation of a fertilized oocyte in the cervix, which can lead to a high risk of complications, such as hemorrhage or uterine rupture requiring urgent medical interventions. This is why a prompt and accurate diagnosis, followed by safe and effective treatment, is of great importance.

As far as the risk factors for cervical pregnancy are concerned, the authors of a recent publication that reviewed the risk factors of ectopic pregnancies in over 90,000 ongoing pregnancies in women undergoing assisted reproductive technology concluded that the main risk factors include two or more previous pregnancies, two or more previous miscarriages, and two or more previous curettages and smoking [2]. Interestingly, they did not observe a positive correlation between the incidence of cervical pregnancy and the history of cesarean section and other types of ectopic pregnancies. Other publications seem to confirm these findings [3].

Diagnosis involves a combination of clinical symptoms, serology, and ultrasound. The most common symptom of CP is vaginal bleeding, which is often profuse and painless but characteristically occurs after a period of amenorrhea [4]. Nonetheless, there are reports of patients presenting with pain and cramps [5]. Clinical diagnostic criteria for the diagnosis of CP include painless vaginal bleeding occurring after amenorrhea, an enlarged cervix compared to a small uterus, and a partially open external os in gynecological examination [5].

Historically, the diagnosis of CP was based on the above-mentioned clinically practical criteria—especially vaginal bleeding without cramping and a soft and enlarged cervix with closed internal and partially open external cervical os [6]. Nowadays, the diagnostic criteria include a combination of clinical findings and ultrasound imaging. As far as the ultrasound examination is concerned, the following findings help to establish the diagnosis:

(1) Presence of a gestational sac with or without cardiac activity in the cervix (below the level of the internal os);
(2) Cervical enlargement with a characteristic barrel shape;
(3) Absence of a sliding sign (no sliding of the gestational sac against the cervical canal when applying pressure);
(4) Presence of blood flow around the gestational sac on Doppler [7].

The standard of management for CP is still under detailed investigation, and there is no clear consensus on a stepwise approach algorithm. Expectant treatment with monitoring of βHCG and regular sonographic examination might be proposed in very early and stable patients; however, the available data on the clinical outcome of this strategy is scarce [8]. A recently published paper on CP management proposed an algorithm based on a patient’s status and timing of the diagnosis [9]. According to Albahlol, hemodynamically stable patients allow more conservative procedures (conservative medical treatment, sonographic-guided aspiration and injection of medications, high-intensity-focused ultrasound, laser photocoagulation, uterine artery embolization, conservative surgery), whereas unstable patients require more radical interventions—including surgery. The author of the article underlines that the final choice of the strategy should always be made based on the individual patient’s condition as well as on the center’s experience and healthcare service availability.

From the above-mentioned therapeutic methods, super-selective uterine artery embolization (UAE) and the use of methotrexate (MTX) have emerged as effective and minimally invasive options in recent years [10]. Traditionally, uterine artery embolization was performed in gynecological patients with heavy vaginal bleeding due to cervical cancer [11]. Other established indications for uterine artery embolization include uterine fibroids, treatment of anticoagulant-associated abnormal uterine bleeding, symptomatic adenomyosis, and ectopic pregnancies [12,13,14].

Advances in invasive radiology have enabled the precise and selective identification of uterine arteries and pathological vessels within cervical ectopic pregnancies, enabling surgery to be performed while controlling bleeding [15]. It has been shown that combined methotrexate therapy together with uterine artery embolization is a more effective method than uterine artery embolization alone, resulting in a shorter duration of hospitalization and fewer complications [16,17]. Additionally, uterine artery embolization has proven effective in managing cervical ectopic pregnancies, with the goal of preserving fertility [17].

The aim of our article is to present our center’s experience and provide a detailed analysis of the available evidence evaluating the efficacy of UAE in the treatment of cervical ectopic pregnancy, providing a comprehensive understanding of this method and its impact on improving patient care in these difficult cases.

## 2. Materials and Methods

### 2.1. Study Design

In this single-center and retrospective study, we evaluated patients with cervical pregnancies (CPs) who were admitted to the Department of Gynecology and Obstetrics and were further referred for endovascular embolization at the Department of Interventional Radiology from 2017 to 2024. The local institutional ethical committee approved this study, and it was performed in accordance with the Helsinki Declaration. All patients gave their informed consent for participation in this study (it was collected either before the endovascular treatment or during the follow-up). All procedures were performed after a multidisciplinary discussion (multidisciplinary boards consisted of gynecologists, obstetricians, and interventional radiologists specialized in gynecological procedures) and consultation with each patient, during which the potential risks and benefits of uterine artery embolization, as well as other therapeutic strategies, were explained.

The inclusion criteria included (1) age > 18 years, (2) CP diagnosed with transvaginal ultrasound (TVUS) and/or magnetic resonance imaging (MRI) with or without fetal heart rate (FHR), and (3) increased levels of serum HCG. The exclusion criteria included (1) CP treated with other methods (surgical or non-surgical) and (2) lack of informed consent, and/or clinical follow-up. Clinical records and diagnostic and procedural findings, as well as follow-up results, were collected and evaluated.

**Figure 1:**
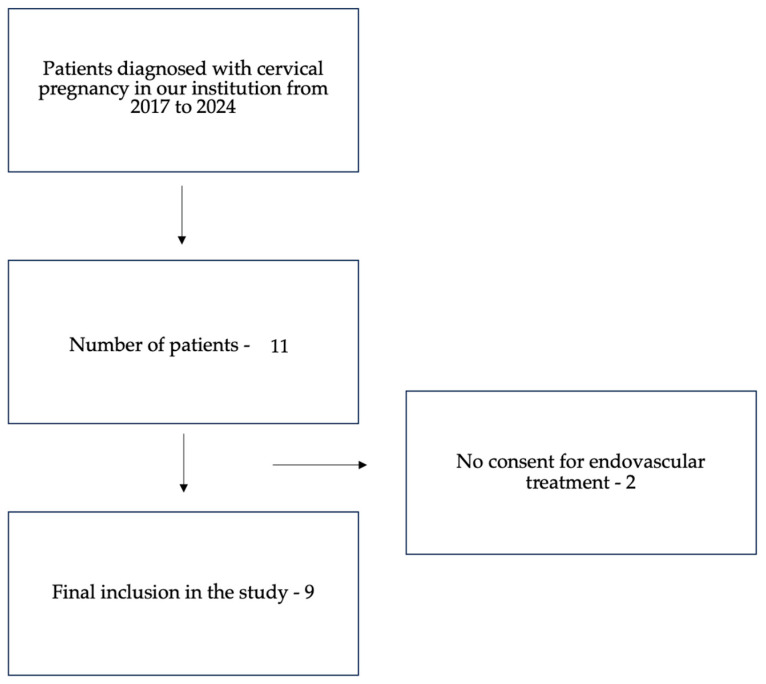
Flowchart showing the patients’ inclusion.

Figure 1 presents a flowchart showing the patients’ inclusion.

### 2.2. Imaging and Diagnosis

**Figure 2:**
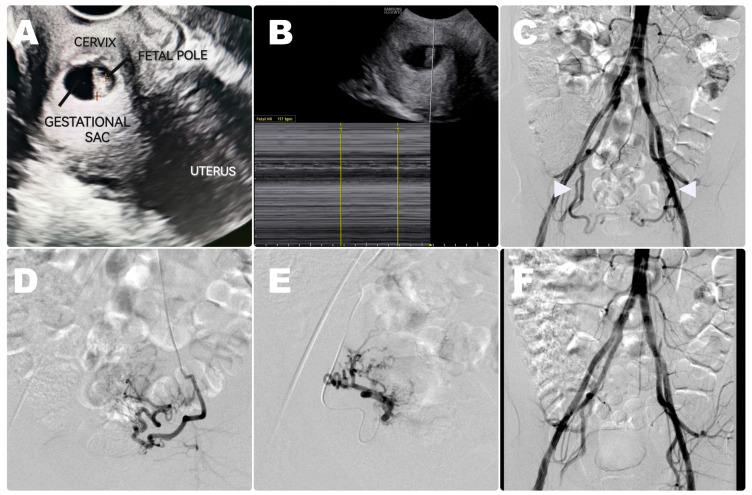
Case of a 33-year-old patient presenting with vaginal bleeding. Transvaginal ultrasound examination suggested a diagnosis of cervical pregnancy (**A**). The presence of a fetal heart rate was confirmed on ultrasound examination (**B**). The patient qualified for endovascular treatment. The initial aortography showed blood supply originating from uterine arteries (white arrows) with no collateral flow from ovarian arteries (**C**). Afterward, selective catheterization of uterine arteries was performed, and embolization with methotrexate and gelfoam was carried out (**D**,**E**). The final angiography showed no flow to the gestational sac (**F**).

In all cases, the diagnosis of cervical pregnancy was confirmed by two independent sonographers by transvaginal ultrasound with Doppler with the following ultrasonographic criteria for CP: (1) endometrial visualization, (2) empty uterine cavity, (3) gestational sac or trophoblast within the cervix, (4) hourglass-shaped (“figure-eight”) uterus with balloon-shaped cervical canal, (5) circular blood flow around the gestational sac, and (6) absence of a “sliding sing”. For the typical diagnosis of cervical pregnancy in ultrasound examination, see Figure 2, panels 1 and 2.

### 2.3. Endovascular Procedure

All interventions were performed by interventional radiologists with more than 5 years of experience in endovascular embolization. The procedures were conducted in an inpatient regimen with an ALARA (as low as reasonably achievable) principle—short fluoroscopy pulses, optimization of X-ray tube and detector position, tight collimation, etc. Under local anesthesia, femoral access was obtained, and a vascular sheath was introduced. Then, a pelvic arteriogram was acquired with a diagnostic catheter placed above the aortic bifurcation in order to depict the anatomy and blood supply to the targeted area. Afterward, internal iliac arteries were selectively catheterized (depending on the vessel caliber with a 4 or 5Fr guiding catheter) and a microcatheter (2.4Fr with a 0.018″ lumen) was advanced coaxially into the horizontal portion of the uterine artery. A total dose of 50 mg methotrexate was administered bilaterally until complete obliteration of the vascular supply to the gestational sac was observed in control angiography. According to our center’s protocol, the first half of the dose of methotrexate in liquid form was slowly injected (60–90 s), and the second portion was mixed with gelfoam and contrast medium—the amount of gelfoam should have allowed stasis in the artery but, at the same time, been low enough to be injected freely through the microcatheter. In the case of pain complaints, analgesic treatment was administered intravenously.

A typical endovascular procedure is presented in Figure 2.

Once the procedure was finished, the vascular sheath was removed, and the puncture site was compressed. All the patients were then transferred to the gynecological ward, where they remained under surveillance for at least 24 h. Additional intravenous analgesic treatment was given if needed.

### 2.4. Gynecological Protocol

Twenty-four hours after intra-arterial methotrexate infusion, the absence of vascularity of cervical pregnancy (CP) was confirmed on ultrasound, and suction curettage was carried out. After performing these procedures, all the patients were monitored in the hospital for another 2–3 days. In terms of uneventful hospitalization, the patient was discharged home. If needed, the patient stayed at the hospital. During short-term follow-up (immediately after the procedure and 30 days after), complications of the management, normalization of menstrual cycle, and β-hCG levels, as well as hemoglobin levels, were assessed. Then, the patients were regularly contacted in order to assess long-term outcomes (structured telephone interview carried out by a physician).

### 2.5. Follow-Up

Both angiographic (endovascular technique, complications) and clinical (vaginal bleeding, menstruation function, recovery) outcomes were measured. Whereas procedural success was defined as the disappearance of uterine arterial flow on the final angiography immediately after the embolization, clinical success was defined as no signs of FHR and/or signs of active vaginal bleeding on gynecological examination.

## 3. Results

In the study period, 11 patients with cervical pregnancies were treated in our center and were preliminarily enrolled in this study. However, two of them were treated with different methods as they did not give their consent for endovascular embolotherapy. Eventually, a total of nine patients (mean age of 36.7 years; range from 25 to 41) were referred for uterine artery embolization (UAE) and included in our study (see the flowchart in Figure 1). As far as the clinical presentation was concerned, all of the reviewed patients complained about abnormal vaginal bleeding resulting in a drop in hemoglobin levels at the time of admission. In all cases, a diagnosis of ectopic cervical pregnancy was suspected by the increased levels of serum β-hCG (ranging from 12,238 to 48,034 mIU/mL), and the findings were further confirmed on transvaginal ultrasound (see Figure 2, panels 1 and 2). The majority of patients (7/9, 78%) had a history of previous pregnancies (ranging from one to four). Diagnostic imaging showed the presence of a gestational sac (size from 12 to 38 mm) and a fetal heart rate (FHR) in six out of nine patients (67%). Estimated gestational ages ranged from 6 to 11 weeks, and all pregnancies except for one were spontaneous (one patient underwent in vitro fertilization).

In terms of endovascular treatment, endovascular procedures resulted in the disappearance of uterine arterial flow on bilateral iliac arteriography. No periprocedural complications were noted. In one case, a postprocedural self-resolving groin hematoma was noted. It did not require further surgical management.

Suction curettage was performed within 24 h after embolization. Blood loss ranged from 50 to 150 mL. Control Doppler examination performed at least 24 h after the embolization confirmed the disappearance of the flow signal around the gestational sac and arrest of the FHR. Apart from mild abdominal pain and slightly raised body temperature (postembolization syndrome), no complications occurred. All the patients were discharged in good clinical condition within 14 days after the embolization. Normalization of both serum β-hCG and hemoglobin levels was observed in all cases.

In short-term follow-up (30 days after the procedure), normal menses resumed in six patients (67%). In another three patients, amenorrhea resolved within 3 months. No long-term complications were noted. The patients’ characteristics are presented in Table 1.

## 4. Discussion

Cervical pregnancy (CP) represents one of the least common types of ectopic pregnancies, with an estimated incidence varying from 1 in 1000 to 1 in 18,000 pregnancies [18,19]. Despite significant advances in diagnosis and treatment, it is still associated with high morbidity and mortality and remains a clinical challenge due to its rarity [20]. As for the predisposing factors, a history of dilatation and curettage, caesarean delivery, and in vitro fertilization, as well as Asherman’s syndrome, are known to increase the risk of CP [21]. In terms of clinical manifestation, it is often uncharacteristic, and the most common symptoms include amenorrhea and uterine bleeding with or without pelvic pain [22]. In our observation, mild vaginal bleeding leading to a slight decrease in hemoglobin levels was the most common presenting symptom.

The diagnosis of CP is based upon ultrasound examination—a gestational sac below the internal os of the cervix with a hypervascular trophoblastic ring indicates a cervical ectopic pregnancy [23]. Apart from this, other classical sonographic findings include an “hourglass cervix” (small uterus with a disproportionately large cervix and a narrowing at the internal os) and a “sliding sac sign” (indicating movement of the gestational sac against the endocervical canal) [18,24]. Modern ultrasound scanners enable a diagnostic accuracy of approximately 90% [24]. In ambiguous cases, magnetic resonance imaging (MRI) might be used for final diagnosis [25]. According to some authors, MRI has several potential advantages, especially in second-trimester CP, when an accurate diagnosis by ultrasound becomes more challenging: a larger field of view and better soft tissue contrast for visualization of the placenta and other tissues (particularly on T2-weighted images). Nonetheless, the existing literature on the use of MRI in the diagnosis of cervical pregnancy is relatively sparse. As far as our observations are concerned, a transvaginal ultrasound was sufficient for making an accurate diagnosis in all cases.

As for the treatment, there are several therapeutic options for CP that can be divided into three main categories: surgical excision, reduction of blood supply, and chemotherapy. All of the above-mentioned options have their advantages and disadvantages, as well as specific indications. Nonetheless, the optimal therapeutic paradigm is not yet established and is often based on the center’s experience and expertise, as well as the individual condition of the patient. It is worth mentioning that the majority of patients with a cervical pregnancy are of low parity, and this is why the current therapeutic trend is to preserve their reproductive ability [26]. This is why traditional hysterectomy was gradually replaced by less invasive and fertility-preserving therapies, including systemic or local methotrexate injection, hysteroscopy, and uterine artery embolization [27,28]. However, minimally invasive forms of hysterectomy (e.g., vaginal hysterectomy) might be considered in selected patients. Alammari et al. presented a case of a 39-year-old multigravida patient with several domestic problems who underwent a successful vaginal hysterectomy for cervical pregnancy [29]. The authors underlined that the benefits of this surgical technique include immediate control of the uterine vasculature and no need to reposition and prepare the patient for the laparotomy. Nonetheless, they concluded that even in patients who have completed childbearing, conservative options must be considered in patients diagnosed with cervical pregnancy.

Concerning the more conservative therapeutic strategies, methotrexate injections are often used as a first-line treatment as they were reported to be an appropriate option in hemodynamically stable women [30,31]. There are no clear recommendations on the methotrexate dosage (both single-dose and multidose intramuscular injection protocols of 50 mg/m2 of body surface area are reported to be effective), but confirmation of normal liver and renal function prior to therapy is advised [31]. Regardless of the implemented protocol, this kind of treatment requires regular monitoring of serum beta-hCG levels in order to ensure an adequate response. This is why it is of great importance to educate patients and ensure they cooperate and adhere to follow-up requirements prior to considering this method of treatment. With a single-dose methotrexate injection, the target beta-hCG decrease is 15% around day 7 after the injection [30]. In the case of an inadequate decline in beta-hCG levels, an additional dose is advised. In terms of clinical success, its success rate in the treatment of ectopic pregnancies is reported to range between 70% and 95%, with higher efficacy among patients presenting with lower baseline serum β-hCG concentrations [31]. However, its main disadvantage is the possibility of a life-threatening bleeding before or after pregnancy evacuation.

As far as the interventional treatment is concerned, there are several options for patients with cervical pregnancy, which include tamponade with a Foley catheter, reduction in blood supply (vessel ligation or embolization), surgical removal of the trophoblast, and intra-amniotic feticide. Tamponade with a Foley catheter (inflated with 20–30 mL saline and placed above the external os) is used in combination with other techniques, e.g., curettage. Ligation of uterine arteries or even internal iliac arteries was reported to be successful but has now been largely replaced by less-invasive endovascular embolotherapy [32,33]. As for the surgical removal of the trophoblast, curettage is the most commonly used technique [34]. This technique is especially efficacious in first-trimester cervical pregnancies with a very high rate of successful terminations and a low rate of complications. Finally, there is a possibility to perform an ultrasound-guided intra-amniotic instillation of potassium chloride [35].

These minimally invasive procedures aid in the preservation of reproductive function, which results in a high rate of intrauterine pregnancy after surgery [36,37]. Stabile et al. described their experience with non-tubal ectopic pregnancies (including six cervical pregnancies) treated with different methods and concluded that a hysteroscopic approach alone or combined with systemic MTX should be a first-line treatment in patients with β-hCG serum levels > 5000 UI/mL [38]. The authors underlined that this method enables precise resection, complete eradication, and minimal blood loss. Nonetheless, it is worth mentioning that this study was conducted in a highly specialized center with experienced surgeons, which might be a limiting factor.

With regard to uterine artery chemoembolization (UAC), its efficacy and safety in the treatment of ectopic pregnancy have been demonstrated by several authors [22,39]. In our institution, it is routinely performed in cesarean scar pregnancies in both elective and emergency cases [40,41,42,43]. It has also been successfully used as a treatment for cervical pregnancies [10,15,44,45,46,47]. Similar to our findings, several authors report that UAC might be a safe and effective method of treatment for CP patients. They conclude that UAC has an acceptable rate of clinical success comparable with other methods, and its main advantages are high efficacy of hemorrhage control and short hospitalization. On the other hand, Martinelli et al. described a case in which a patient had to undergo a hysterectomy after embolization due to ischemic degeneration of a concomitant myoma [48]. This is why the choice of the most appropriate treatment of a patient with cervical pregnancy should always be made after careful evaluation of clinical and imaging examination, thorough discussion with the patient, and the center’s experience.

Our study has the following limitations. First and foremost, it is a retrospective and single-center study with a relatively small number of included patients, which impacts the generalizability of our findings. Secondly, due to the fact that uterine artery embolization became a first-line therapy for cervical pregnancies in our center, our study lacks a control arm showing the results of patients treated with different methods.

## 5. Conclusions

In summary, uterine artery chemoembolization with methotrexate administration followed by suction curettage appears to be a safe, minimally invasive, and effective procedure for patients with ectopic cervical pregnancy. In addition to this, it results in a high rate of vaginal bleeding control and a low complication rate. Nonetheless, due to the limitations of our study, further research is needed in order to draw firm conclusions.

## Figures and Tables

**Table 1:** Patients’ characteristics and clinical outcomes.

Age on Admission (Years)	GA (Weeks)	GS Size (mm)	MTX Used (mL)	BHCG Normalization (Weeks)	FHR	Complications
1. 38	6	12	50 mL	3	−	None
2. 37	7	15	50 mL	3	+	None
3. 41	8	15	50 mL	4	+	None
4. 33	8	23	50 mL	4	+	Groin hematoma
5. 25	6	13	50 mL	2	−	None
6. 38	7	17	50 mL	5	−	None
7. 39	11	25	50 mL	6	+	None
8. 39	11	38	50 mL	6	+	None
9. 40	7	17	50 ml	3	+	None

## Data Availability

Data is available upon request from the Corresponding Author.

## Abbreviations

The following abbreviations are used in this manuscript:

CP	Cervical pregnancy
UAE	Uterine artery embolization
TVUS	Transvaginal ultrasound
MRI	Magnetic resonance imaging
